# Supplementary material for: Wide-field calcium imaging of cortical activation and functional connectivity in externally- and internally-driven locomotion
Source: Res Sq. 2023 Apr 14:rs.3.rs-2776902. Preprint. [Version 1] doi: 10.21203/rs.3.rs-2776902/v1 (PMC10120783; doi:10.21203/rs.3.rs-2776902/v1)
Supplement: 1 [file NIHPPRS2776902V1-supplement-1.pdf]

Supplementary Tables

Supplementary Table 1. Warning tone parameters.

Each tone consisted of 3 pitches in sequence, played for 310 ms with a 23 ms silent period in between to increase tone salience, for a total of 1.0 s. Different pitches were played to distinguish between the upcoming transitions.

**Upcoming transition**	**Pitch in Hz**		
	1st pitch	2nd pitch	3rd pitch
starting	10,000	10,000	10,000
stopping	4,000	4,000	4,000
accelerating	4,000	(silence)	10,000
decelerating	10,000	(silence)	4,000
maintaining	7,000	(silence)	7,000

Supplementary Table 2. PLSR metrics.

**Metric**	**Regression type**	**Mean**	**Std. dev. across mice**	**Std. dev across regressions**
# PLSR components	*parameters*	**1.8**	0.6	1.0
	periods	**4.7**	0.8	1.8
Mean squared error	*parameters*	**0.95**	0.02	0.05
	periods	**0.38**	0.14	0.27

Supplementary Table 3. **Network connectivity percent variance explained per behavior variable.**

**Behavior variable**	**Mean**	**Std. dev. across mice**	**Std. dev across regressions**
Speed	9.5%	2.1%	6.3%
Acceleration	6.4%	1.7%	2.8%
Duration	8.2%	1.7%	7.7%
Pupil diameter	9.8%	4.4%	2.2%
Behavior periods	16.8%	2.7%	10.1%

Supplementary Figures

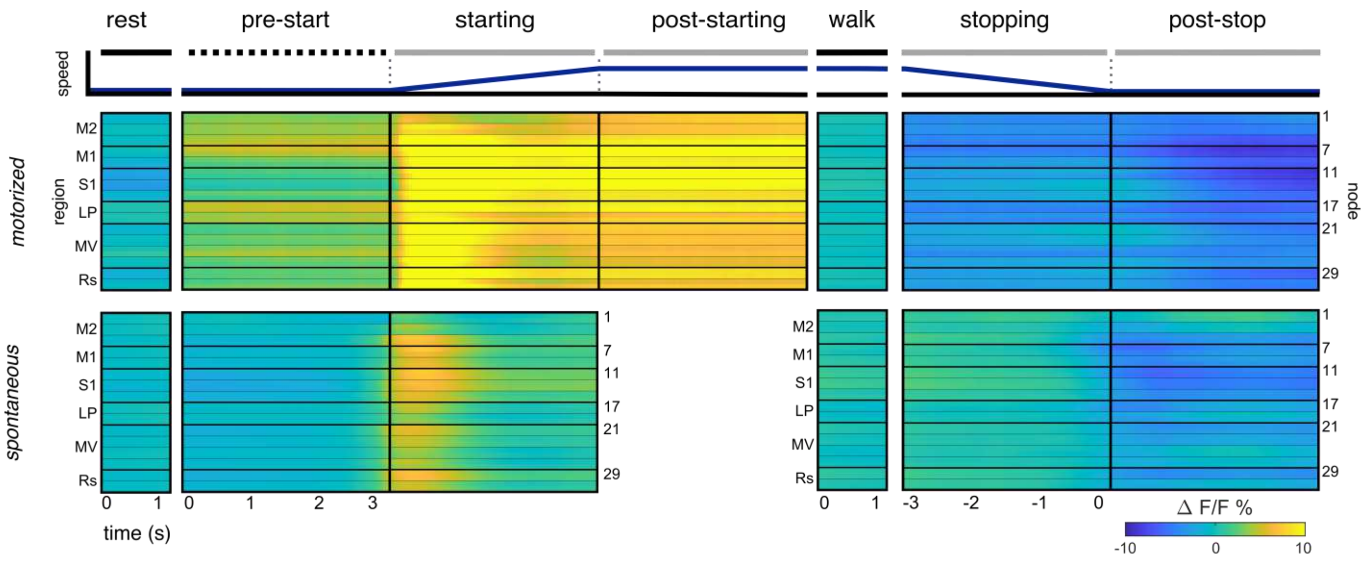

Supplementary Figure 1. Activation in motorized treadmill and spontaneous locomotion

ΔF/F% of nodes across locomotion behavior period for motorized treadmill (top) and spontaneous locomotion (bottom) conditions. There is no post-starting period defined in the spontaneous condition.

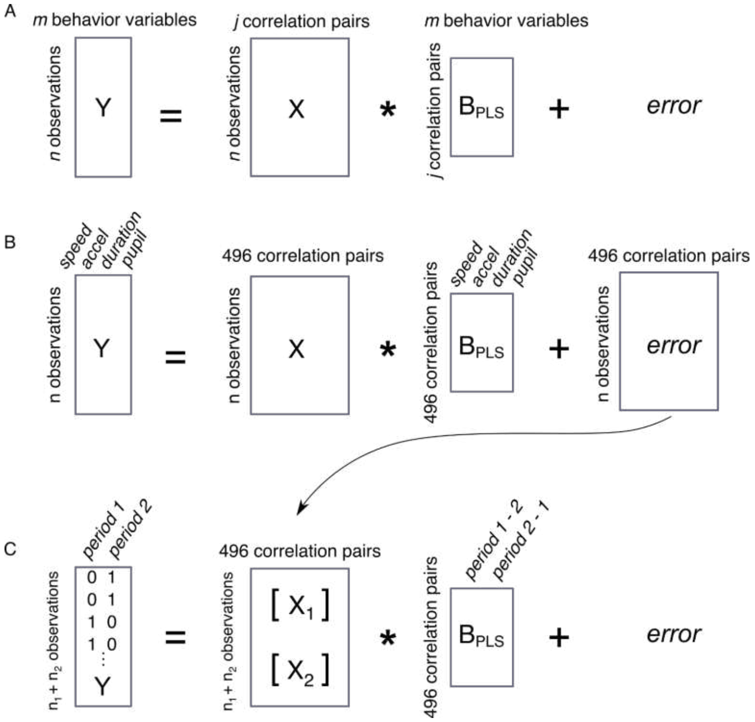

Supplementary Figure 2. Diagram of partial least squares regression (PLSR) steps.

A. Diagram of the general regression equation used. **B.** Diagram of the equation used for each behavior period regressing behavior parameters against FC. The error term (residuals) of these regressions is then carried forward as the node-node correlation data in the regressions across behavior periods (the X matrix in C), as indicated by the arrow. **C.** Regression equation used to calculate the change in FC across behavior periods.

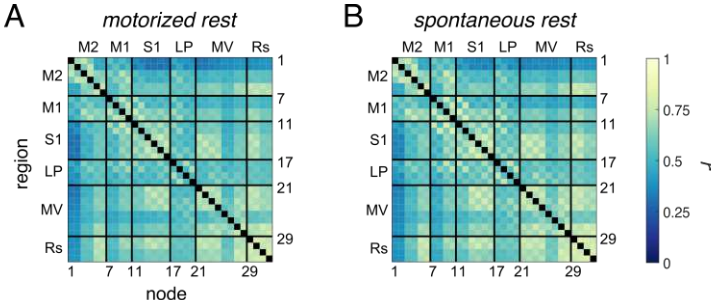

Supplementary Figure 3. Average correlations during rest in the spontaneous locomotion condition.

A. The correlation between node pairs during rest in the spontaneous locomotion condition, averaged across mice. **B**. The correlations shown in A with the Fisher transformation applied.

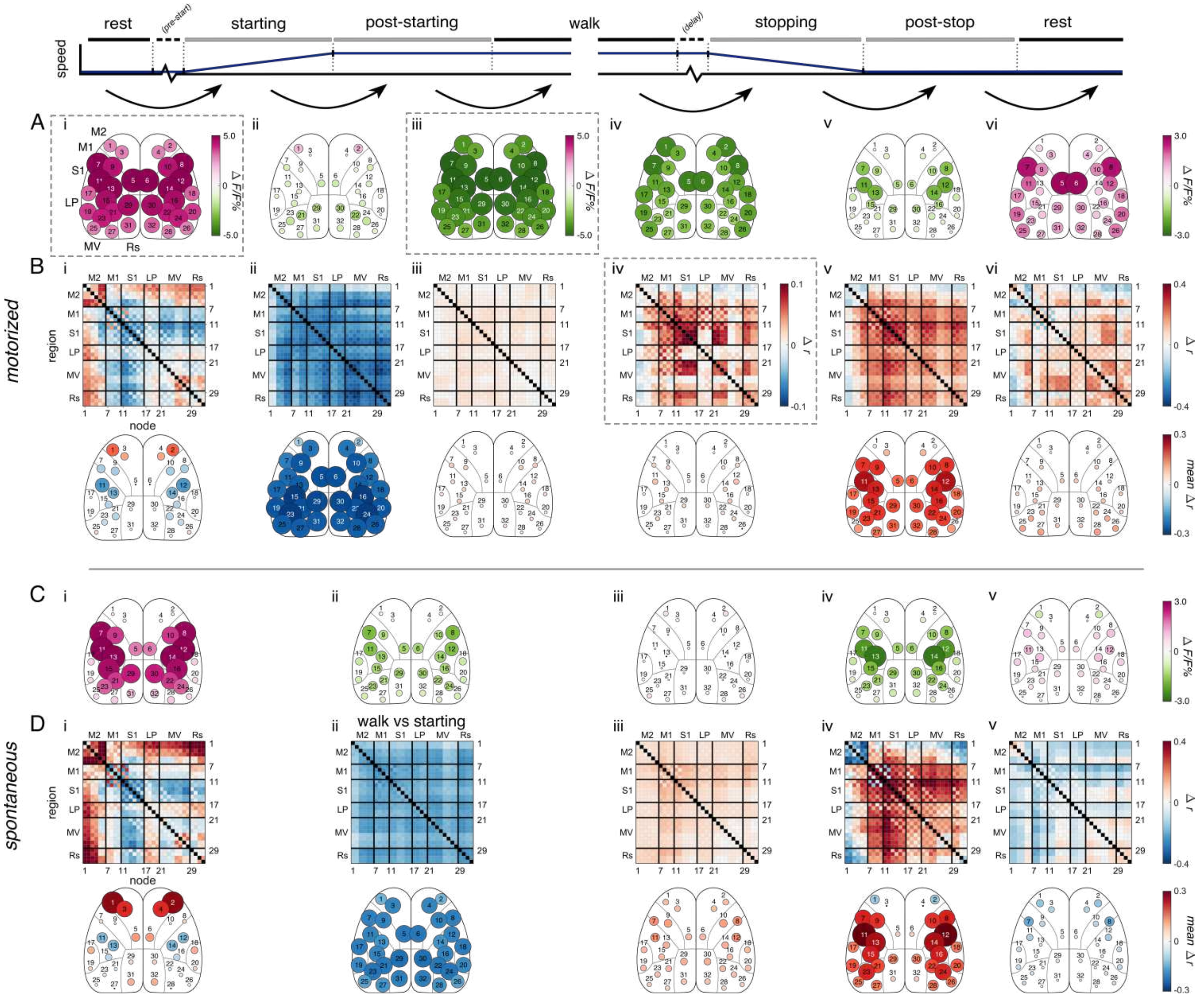

Supplementary Figure 4. Change in correlation across sequential behavior periods.

A & B. Significant changes in fluorescence (A) and correlations (B) across sequential behavior periods in the motorized treadmill condition, calculated and displayed as in Figures 2 & 3. Directly above each correlation matrix, the black arrow indicates the adjacent behavior periods being compared (i-vi). Positive values indicate increases in fluorescence or correlation across the change in behavior period. Delay periods were not included here. **C & D.** Similar to A & B, but for the spontaneous locomotion condition. Note that the spontaneous condition has no post-starting period, so the change from starting to walk (Cii & Dii) is provided instead.

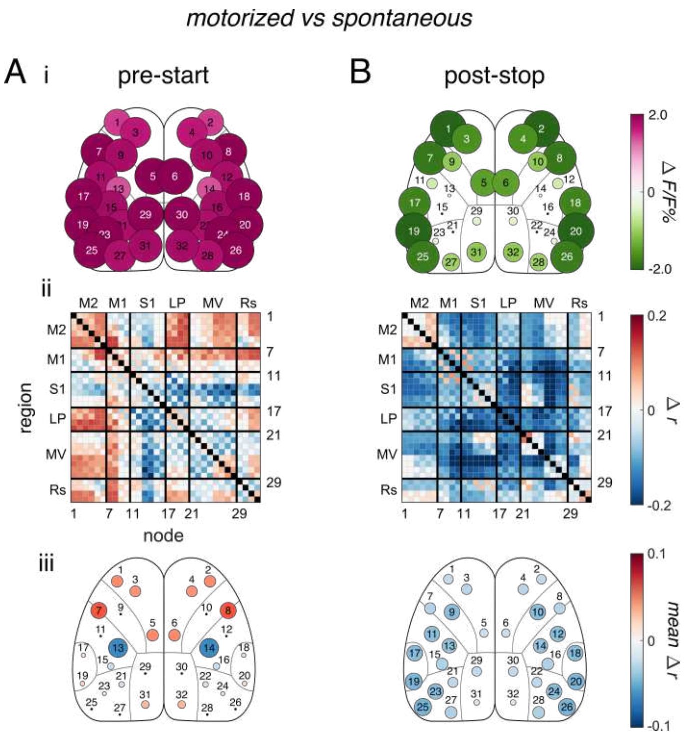

Supplementary Figure 5. Direct comparisons of pre-start and post-stop periods across conditions.

A. Significant difference in pre-start period fluorescence and FC between motorized and spontaneous locomotion conditions (motorized minus spontaneous), calculated and displayed as in Figure 3. **B**. Similar to A, but for post-stop periods.

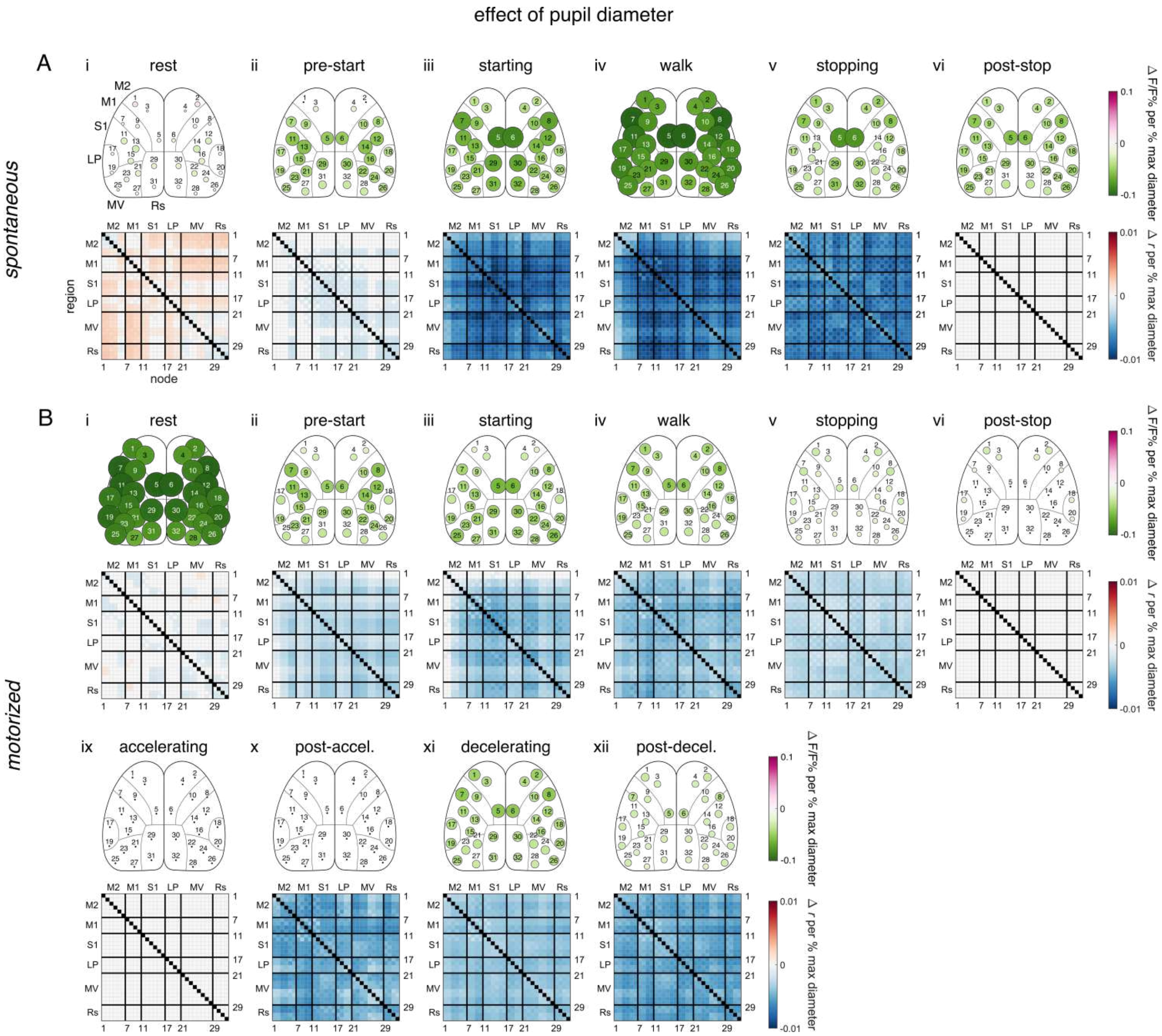

Supplementary Figure 6. Effect of pupil diameter on behavior periods.

A. Significant changes in fluorescence and correlation per % of max pupil diameter for spontaneous periods (α<0.05, permutation test with false discovery rate correction). **B**. Similar to A, but for motorized periods. Data in all panels are calculated and displayed as in Figure 2.
